# Uncovering DENV, CHIKV, and ZIKV in Urban Wastewater in Brazil Through Genomic and Molecular Screening

**DOI:** 10.3390/microorganisms13092164

**Published:** 2025-09-17

**Authors:** Juliana Calabria de Araujo, Ana Paula A. Carvalho, Talita Adelino, Felipe Campos M. Iani, Natalia Rocha Guimaraes, Sara Candida F. Santos, Cintia D. Leal, Manuelle Natividade, Mauricio Lima, Mariana Almada, Ana Carolina Bertuce, Augusto Guerra, Maria Cristina M. Costa, Flavia Saia, Vagner Fonseca, Marta Giovanetti, Livia V. Frutuoso, Luiz Carlos Junior Alcantara

**Affiliations:** 1 Department of Sanitary and Environmental Engineering, Universidade Federal de Minas Gerais-UFMG, Belo Horizonte 31270-901, Brazil; assadanapaula@gmail.com (A.P.A.C.); cintia@desa.ufmg.br (C.D.L.); carolcbertuce@gmail.com (A.C.B.); 2 Laboratório Central de Saúde Pública, Fundação Ezequiel Dias, Belo Horizonte 30510-010, Brazil; talita.adelino@funed.mg.gov.br (T.A.); felipe.iani@funed.mg.gov.br (F.C.M.I.); natyroguiman@yahoo.com.br (N.R.G.); saracandidaufmg@gmail.com (S.C.F.S.); maurili15@hotmail.com (M.L.); 3 FarGenix—Pharmacogenomics Laboratory, Department of Social Pharmacy, Universidade Federal de Minas Gerais-UFMG, Belo Horizonte 31270-901, Brazil; manuellenatividade@gmail.com (M.N.); augustoguerramg@gmail.com (A.G.); 4 Centro Federal de Educação Tecnológica—CEFET-MG, Belo Horizonte 30510-000, Brazil; marisilvalmada@gmail.com (M.A.); cristinacosta.cefetmg@gmail.com (M.C.M.C.); 5 Departament of Marine Sciences, Universidade Federal de São Paulo—UNIFESP, Santos 11070-100, Brazil; ft.saia@unifesp.br; 6 Centre for Epidemic Response and Innovation (CERI), School for Data Science and Computational Thinking, Stellenbosch University, Stellenbosch 7600, South Africa; vagner.fonseca@gmail.com; 7 Department of Exact and Earth Sciences, University of the State of Bahia, Salvador 40170-010, Brazil; 8 Department of Sciences and Technologies for Sustainable Development and One Health, Universita Campus Bio-Medico di Roma, 00128 Roma, Italy; giovanetti.marta@gmail.com; 9 Instituto René Rachou, Fundação Oswaldo Cruz, Belo Horizonte 21040-900, Brazil; alcantaraluiz42@gmail.com; 10 Coordenação Geral de Vigilância de Arboviroses, Departamento de Doenças Transmissíveis, Secretaria de Vigilância em Saúde e Ambiente, Ministério da Saúde, Brasília 70058-900, Brazil; livia.vinhal@saude.gov.br

**Keywords:** arbovirus, genomic surveillance, molecular screening, RT-qPCR diagnostic kit, public health laboratories, wastewater surveillance

## Abstract

This study evaluated and compared molecular methods (Whole Genome Sequencing-WGS, MinION, and RT-qPCR) for the detection of arboviruses Dengue (DENV), Chikungunya (CHIKV), and Zika (ZIKV) in 63 hospital and municipal wastewater samples collected from July 2022 to May 2023 in the region of Belo Horizonte, Brazil. Detection rates varied substantially across the methods (WGS, MinION, and RT-qPCR). DENV was identified in 24% (15/63) of samples using a hybrid capture method of WGS and MinION sequencing and in 66.6% (20/30) using only WGS but was not detected using the CDC Trioplex RT-PCR Assay Kit or ZDC (IBMP). CHIKV was detected in 19.0% (12/63) of the samples by WGS and MinION and in 85.7% (12/14) using only MinION sequencing. Using the RT-qPCR kit to detect CHIKV yielded a rate of 4.7% (3/63) in false positives. ZIKV was found in only one sample (1/63) by WGS, while RT-qPCR yielded a high false positive rate (65.1%, 41/63). These findings highlight the operational advantage of these methods (WGS and MinION) for enhancing early-warning surveillance where standard RT-qPCR might underperform in low-prevalence settings. This is the first study that has compared these methods to detect and genetically characterize DENV, CHICK, and ZIKV in wastewater in Brazil and has indicated that hospital wastewater can be used as a sentinel system for arbovirus surveillance. The relative effectiveness of genomic wastewater surveillance for arboviruses was demonstrated, and it was found that diagnostic RT-qPCR kits used for clinical samples were not directly suitable for environmental surveillance. The feasibility of arbovirus wastewater surveillance as an epidemiological tool was demonstrated, although absolute quantifications were not performed.

## 1. Introduction

Arboviruses such as Dengue virus (DENV), Chikungunya virus (CHIKV), and Zika virus (ZIKV) continue to pose serious public health threats across tropical and subtropical regions, particularly in Brazil. In recent years, the country has experienced increasingly severe outbreaks of arboviral diseases, with significant impacts on healthcare infrastructure and population health. By May 2024, Brazil had recorded over 4.2 million probable cases of Dengue—the highest ever reported in a single year—with more than 2900 confirmed deaths. During the same period, Chikungunya accounted for over 250,000 probable cases, with a marked increase in co-circulation of arboviruses in several regions, particularly in the Southeast and Central-West [1]. These numbers highlighted the urgent need for continuous and early-warning surveillance tools, such as wastewater surveillance.

Traditional epidemiological surveillance systems—primarily based on clinical and laboratory-confirmed cases—are essential for outbreak response but often suffer from reporting delays, underdiagnosis, and limited coverage, particularly in areas with poor healthcare access. In this context, Wastewater Environmental Surveillance (WES), the systematic monitoring of pathogens in wastewater and water, has emerged as a promising complimentary epidemiological tool for early detection and community-level monitoring of pathogens. WES involves monitoring the presence and concentration of viral RNA or DNA in wastewater and environmental samples to infer the circulation of infectious agents within the population [2]. This approach is especially relevant for diseases with asymptomatic or subclinical presentations, such as Zika and mild Dengue cases, which frequently go unreported in clinical systems.

However, the application of WES for arboviruses remains limited by several technical and methodological challenges. One of the main limitations is the typically low viral load in environmental matrices, necessitating the use of highly sensitive detection methods such as reverse transcription quantitative polymerase chain reaction (RT-qPCR) [3]. Although RT-qPCR is widely regarded for its sensitivity, performance can be susceptible to inhibitors present in wastewater [4], as well as by RNA degradation and genetic variability [5]. Additionally, detection efficiency is influenced by the viral shedding rate, which varies across individuals and viruses. For instance, Chen and Bibby [6] emphasized that successful detection of ZIKV depends not only on method sensitivity but also on a sufficiently high rate of viral shedding in excreta.

While WES has proven successful for fecal-shed viruses like SARS-CoV-2 [7,8,9], human adenovirus, norovirus, rotavirus, hepatitis A virus, and poliovirus [10,11,12], evidence for arboviruses remains scarce. Some viruses, such as the polyomaviruses BKV and JCV, which are shed in urine, are consistently detected in wastewater [13,14], suggesting a potential route for arbovirus detection. Diamond et al. [15] reported the potential of wastewater surveillance, a cost-effective and scalable approach for generating high-resolution health data and highlighted the medium-to-high feasibility of detecting climate-sensitive pathogens such as malaria, Dengue, Zika, and West Nile viruses in wastewater. Nonetheless, studies specific to DENV, CHIKV, and ZIKV monitoring in sewage remain limited. For example, Zhu et al. [5], attempted to detect ZIKV in archived wastewater samples from the 2015–2016 outbreak in Bahia, Brazil, but found no detectable RNA, although the study provided valuable insights into RNA stability and recovery. Chandra et al. [16] investigated the persistence of Dengue (Serotypes 2 and 3), Zika, Yellow Fever, and Murine Hepatitis virus RNA in wastewater matrices and reported that the RNA of these viruses persisted in wastewater over a range of temperature (6, 25, and 37 °C), supporting the potential for WES of arboviral outbreaks. Thakali et al. [17] reported that conducting DENV wastewater surveillance remains challenging due to the absence of validated protocols and standardized wastewater processing methods. On the other hand, Monteiro et al. [18] reported the detection of DENV in 25% and CHIKV in 11% of 273 wastewater samples collected in Portugal (during May 2022 to April 2023) using RT-qPCR, demonstrating feasibility under optimized conditions, but did not correlate viral load in wastewater with number of cases. Additionally, Wolfe et al. [19] reported DENV RNA detection in wastewater solids samples from three WWTP in Miami, FL, USA. They showed that wastewater detection of DENV was possible with as few as 4.23 confirmed dengue cases per 1 million people. Yet, these examples remain isolated, and further investigation into effective, scalable methodologies is urgently needed—particularly in high-burden countries like Brazil.

Building on our previous findings [20], which detected multiple human viruses (Adenoviridae, Astroviridae, Caliciviridae, and Coronaviridae, among others), including Mpox in hospital and municipal wastewater samples [20], this study investigates the detection of arboviruses in wastewater using three molecular approaches: RT-qPCR, hybrid capture method for whole genome sequencing, and Oxford Nanopore’s MinION sequencing. Genomic methods are hypothesized to outperform RT-qPCR (clinical kits) in environmental matrices. We conducted an 11-month surveillance campaign in Belo Horizonte (during 2022 and 2023), the third-largest metropolitan area in Brazil, because it is an area of high arboviral circulation. In fact, the city of Belo Horizonte, a densely populated urban center in southeastern Brazil, experiences recurring outbreaks of arboviral diseases, making it a critical location for improving and validating surveillance techniques. This comparative analysis aims to evaluate the sensitivity and practicality of each method, and to advance the use of WES as an early-warning tool for arbovirus outbreaks in urban settings. We aim to inform the development of robust surveillance frameworks capable of supporting public health interventions and guiding timely responses to arboviral threats.

While molecular detection methods such as RT-qPCR are widely used for virus identification in environmental samples, recent advances in genomic technologies—including nanopore-based whole genome sequencing (e.g., Oxford Nanopore’s MinION)—offer complementary approaches for detecting and characterizing viral genomes directly from complex matrices like wastewater. The aim of this study was to evaluate and compare the effectiveness of different molecular methods (hybrid capture for WGS, MinION sequencing, and RT-qPCR diagnostic kits used for clinical samples) for detecting arboviruses in wastewater. This is the first pilot study that has applied and compared these methods to successfully detect and genetically characterize DENV, CHIKV, and ZIKV in wastewater during a dengue outbreak in Brazil. Our study used wastewater samples from hospitals and community wastewater, and to collect these samples, permissions were obtained from the responsible authorities of the hospitals and the sanitation company.

## 2. Materials and Methods

**Figure 1:**
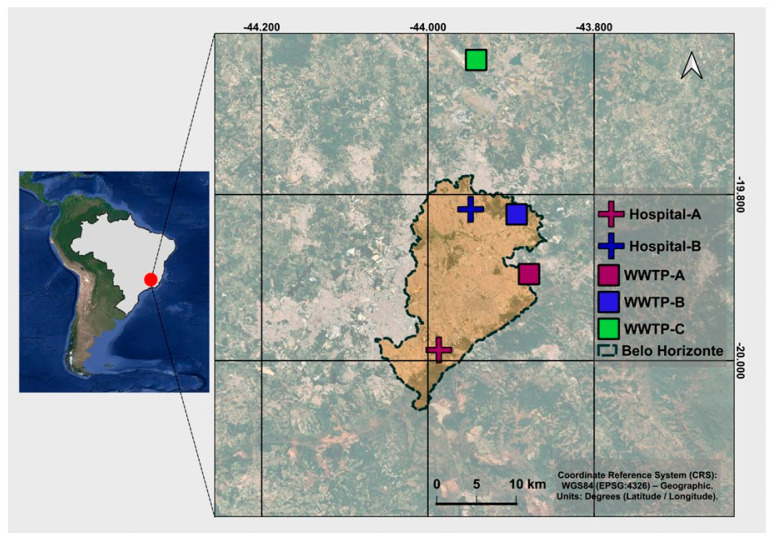
Map of Brazil with the location of Belo Horizonte city and the 5 sampling points identified.

Belo Horizonte is the capital of the state of Minas Gerais, in the Southeast of Brazil, and has a population of 2.7 million people and a metropolitan area with 6 million inhabitants [21]. Sewage samples were collected from five sites in the metropolitan region (Figure 1): two hospitals (designated Hospital A and Hospital B, with Hospital A serving as a reference center for infectious disease treatment in the state), and two municipal Wastewater Treatment Plants (WWTP) that both treat sewage from approximately 2.3 million people, with a mean influent load of 2300 L·s^−1^ for WWTP-A (WWA) and 2089 L·s^−1^ for WWTP-B (WWB). The third WWTP (WWC) receives sewage from an international airport (influent flow of 5.0 L·s^−1^, and population equivalent of 2560 inhabitants). The geographic coordinates for these locations were mentioned in our previous study [20], except for WWTP-C: 19°63′57.1″ S 43°96′69.3″ W (Figure 1).

Sewage samples were collected twice monthly from 23 July 2022 to May 2023. In total, 63 samples were collected and sequenced: 28 from the hospitals (14 samples each), 18 from WWTP-A and 15 from WWTP-B. Additionally, 2 samples were obtained from WWTP-C at the international airport on 15 March 2023. Hospital sewage sampling followed previously established protocols [8], which comprised 3 h-composite samples collected in the morning, representing a total of 6 L. For the WWTPs, 24 h-composite samples were collected at the plant inlets, comprising a total of 10 L. Samples were homogenized, and 1 L aliquot was transported at 4 °C to the lab. Both hospital and community wastewater samples were directly filtered using 0.45 µm electronegative membranes (30 to 50 mL), as previously described [7,8], with minor modifications: MgCl_2_ (2.5 M) was omitted, and pH adjustment to 3.5 was not performed. Viral genetic material was extracted from all the membranes using the AllPrep PowerViral DNA/RNA Kit (Qiagen^®^, Hilden, Germany), following the manufacturer’s instructions. Extracted nucleic acids were resuspended in 100 µL of RNase-free ultrapure water and stored at −80 °C.

All 63 samples underwent whole-genome sequencing through target enrichment using a hybrid capture method. Target enrichment was performed using the Illumina VSP panel, which targets 66 DNA and RNA viruses—including Mpox, Poliovirus, Influenza, Dengue, Chikungunya, Zika, and SARS-CoV-2—following the protocol described previously [20]. The Illumina RNA Prep with Enrichment Indexes Set A (96-sample format; Catalog No. 20026121) and the Illumina VSP panel (Catalog No. 20088154) were used to prepare the sequencing libraries by synthesizing cDNA from the concentrated wastewater samples. Libraries were sequenced on the NextSeq™ 2000 System (2 × 150 bp), generating 8–10 million reads per sample. FASTQ files were analyzed using the Illumina DRAGEN™ Microbial Enrichment pipeline, available via BaseSpace™ Sequence Hub, with default settings for viral detection. Additionally, raw data were analyzed using the Genome Detective software 2.94 [22]. The sequences were deposited in GenBank under the sample codes SAMN42174356 to SAMN42174437.

For some samples (15 selected samples) MinION sequencing was used to confirm the presence of DENV1 and CHIKV. Multiplex PCR was conducted using Q5 Hot Start high-fidelity DNA polymerase (New England Biolabs) and a CHIKV whole-genome sequencing primer scheme (the primers are divided into two separate pools, A and B) [23,24]. For Dengue, samples were processed using CADDE primers and Oxford Nanopore Technologies, following protocols validated in previous studies [25,26].

For DENV, CHIKV, and ZIKV, we used the GoTaq Probe 1-Step RT-qPCR kit (Promega) with the primers and probes of the Trioplex Real-time RT-PCR Assay kit (CDC (https://stacks.cdc.gov/view/cdc/155076/cdc_155076_DS1.pdf, accessed on 4 June 2024), according to the manufacturer’s instructions. For RT-qPCR using Trioplex kit, viral isolates were used as a positive control and water as a negative control. In-house primers were also applied (for CHIKV according to Lanciotti et al. [27], for ZIKV according to Lanciotti et al. [28], and for DENV according to Johson et al. [29]), as well as the ZDC (IBMP) kit, which detects ZIKV, CHIKV, and DENV1–4 through RT-qPCR using hydrolysis probes for specific molecular targets defined by the manufacturer, though the exact genomic regions are not disclosed (https://www.ibmp.org.br/wp-content/uploads/2025/01/INF-047-15-Kit-IBMP-Biomol-ZDC.pdf, accessed on 4 June 2024). For RT-qPCR using ZDC (IBMP), positive controls were provided in the kit by the manufacturer and water was used as a negative control. We sequenced 3 CHIKV- and 4 ZIKV-positive amplified fragments of RT-qPCR to try to confirm the detection of the targets. The amplicons were purified using magnetic beads and subsequently sequenced on the Ion PGM platform (Thermo Fisher Scientific, Waltham, MA, USA), following the manufacturer’s recommendations. Sequences obtained were assembled using SPAdes v3.15.5. The resulting contigs were analyzed with BLAST 2.17.0, and the most significant hits were inspected in AliView to assess whether the diagnostic primers could anneal within the amplified fragments.

Bioinformatics analyses were carried out using standardized workflows. Illumina WGS data were processed through the DRAGEN™ Microbial Enrichment pipeline on the BaseSpace™ Sequence Hub (Illumina, default settings), followed by viral genome assembly and annotation with Genome Detective 2.94 [22]. For MinION sequencing, raw reads were basecalled and demultiplexed using Oxford Nanopore Technologies’ Guppy softwarev. 6.5.7, and consensus genomes were generated and further analyzed with Genome Detective [22]. Amplicons sequenced on the Ion PGM platform (Thermo Fisher Scientific) were analyzed using a custom workflow comprising quality trimming (Trimmomatic), de novo assembly (SPAdes), read mapping (minimap2), alignment processing (Samtools and Bedtools), variant calling (Bcftools), and consensus refinement (Pilon).

## 3. Results and Discussion

As shown in Table 1, the detection frequencies of DENV, CHIKV, and ZIKV varied depending on the method used. Target enrichment for WGS, RT-qPCR, and MinION sequencing was applied to 63 wastewater samples collected from five locations (3 WWTPs and 2 hospitals) over an 11-month period (July 2022 to May 2023). DENV-1 was detected in 15 out of 63 samples (23.8%), ZIKV in only one sample (1.6%) and CHIKV in 12 (19%) (Table 1) using WGS and MinION sequencing. The highest detection frequencies were observed in samples from Hospital A (Table 1). In general, higher frequency of detection was observed in hospital wastewater compared to community wastewater; this is likely due to the fact that samples were collected directly from sewer manholes in the hospitals, being therefore less diluted compared to samples from WWTP. Notably, the only ZIKV-positive sample originated from Hospital A and was collected on 8 February 2023. This finding is interesting, though it should be interpreted with caution, especially considering that the official data for Belo Horizonte showed that there were no confirmed cases of Zika in the city in 2022, 2023, or 2024 [30,31]. It may reflect a background signal or artifact, and does not, on its own, provide sufficient evidence to suggest cryptic or subclinical circulation of ZIKV in the population. In contrast, dengue cases were substantially higher, with 776 and 2874 confirmed cases reported in February and March 2023, respectively [31,32]. For Chikungunya, 404 and 1314 confirmed cases were recorded during the same period [31]. RT-qPCR testing using the Trioplex CDC diagnostic kit—routinely applied to clinical samples—yielded negative results for DENV across all 63 wastewater samples (Table 1). Even when using an alternative diagnostic assay (ZDC kit from IBMP), no DENV-positive samples were identified (Table 2). For CHIKV, 95.2% of the samples (60/63) yielded negative results, while 4.7% (3/63) were false positives (Table 1 and Table 3). For ZIKV, non-specific amplification occurred in 65.1% of the samples (41/63), resulting in a high rate of false positives (Table 1 and Table 4). We considered these false positives because the RT-qPCR-amplified fragments were sequenced and no CHIKV and/or ZIKV sequences were retrieved.

Table 2 and Table 3 present a comparison between two sequencing methods, hybrid capture WGS and MinIOn sequencing and RT-qPCR for the detection of DENV-1 and CHIKV, respectively.

Detection frequencies differed notably between methods. For DENV-1, the hybrid capture WGS method identified 20 positive samples out of 30 (66.6%) whereas MinION sequencing detected only 2 out of 15 samples (13.3%) and there was no detection by RT-qPCR (Table 2). Conversely, for CHIKV, MinION sequencing yielded a much higher detection frequency, in 12 out of 14 samples (85.7%) compared to only 2 out of 30 samples (6.7%) using WGS (Table 3). These differences might be associated with the primers used and the amplification of different regions of the virus genomes in DENV1 and CHIKV. For MinION sequencing, the regions amplified for sequencing for DENV and CHIKV use an overlapping primers technique to capture the entire genome. So, if some primer fails, we could still sequence some fragments. For Illumina, the hybrid capture WGS method uses a viral surveillance panel, and we do not know the regions amplified because it is a commercial panel, and the oligonucleotides region was not mentioned by the company.

Importantly, DENV and CHIKV RNA were first detected in hospital wastewater samples as early as July and August 2022, when the reported number of new dengue and chikungunya cases per epidemiological week in Belo Horizonte were relatively low (Table 2 and Table 3, respectively): 31 and 3 cases (23 July 2022), and 23 and 6 cases (12 August 2022), respectively [31,32]. In contrast, detection in community wastewater (WWTP-A) occurred later, in February and March 2023, coinciding with a substantial increase in reported cases: 267 and 148 cases for dengue and chikungunya (Table 2 and Table 3, respectively), respectively (23 February 2023), and 430 and 253 cases, respectively (1 March 2023) [31,32].

These findings suggest that hospital wastewater can serve as an effective sentinel system for early arbovirus surveillance in urban settings—even during periods of low community case numbers (under 20 cases per week)—by mitigating the dilution effects observed in large-scale municipal sewage systems, where each WWTP services around 1.3 million people. Importantly, the detection of DENV and CHIKV in hospital wastewater highlights its potential as an early warning indicator of community transmission. Because wastewater reflects aggregated viral shedding from both symptomatic and asymptomatic individuals, identifying arboviruses in this setting could alert public health authorities before clinical case counts rise, enabling timely vector control interventions and clinical preparedness.

In the case of ZIKV (Table 4), the only positive sample was from the sewage of Hospital A (in 02/08/2023), which is the reference hospital for infectious diseases in the state of Minas Gerais, and Zika cases were not reported in that municipality in 2022 and 2023, despite having reported cases of Zika in the state of Minas Gerais (20 and 54 cases, respectively, in 2022 and 2023 [30]. Thus, cryptic circulation of ZIKV could be occurring in hospital sewage. The comparison between the hybrid capture WGS method and RT-qPCR for ZIKV is shown in Table 4. ZIKV was detected in only one out of 30 samples (3.33%) using WGS, while RT-qPCR resulted in a high rate of false positives, 22 out of 28 samples (78.6%). The sequencing of the amplified fragments from both diagnostic kits (Trioplex CDC and ZDC-IBMP) confirmed the absence of ZIKV sequences. In one case, the sequencing of the amplicons generated by RT-qPCR—short fragments under 100 base pairs, which constrain more robust analyses—suggested that the amplified products may correspond to bacterial sequences, including species from the *Sulfurospirillum* genus, thus indicating that the positive RT-PCR results may have resulted from non-specific amplification.

The relative sensitivity of the methods varied according to the virus investigated. For DENV-1, hybrid capture WGS detected 66.6% (20/30) of positives, compared with 13.3% (2/15) by MinION and none by RT-qPCR. For CHIKV, MinION sequencing achieved 85.7% (12/14), whereas WGS detected 6.7% (2/30) and RT-qPCR 10% (3/30, subsequently confirmed as false positives). For ZIKV, WGS identified 3.3% (1/30) while RT-qPCR yielded 78.6% (22/28), all false positives. These results demonstrate that sequencing methods consistently outperformed RT-qPCR in wastewater, though performance was virus-specific: WGS was more effective for DENV, and MinION for CHIKV.

Our results suggest that the clinical kits evaluated for arboviruses detection, Trioplex CDC and ZDC-IBMP, are not directly applicable to genetic material extracted from environmental matrices and may generate false positives or negatives. Nonetheless, in a previous study [20], we showed that qPCR diagnostic kits used on clinical samples for the detection of Mpox (five PLEX assays diagnostic kits from Bio-Manguinhos), and RT-qPCR assays for Influenza A, Influenza B and SARS-CoV-2 (developed by Bio-Manguinhos) performed well for monitoring wastewater samples.

It is also important to note that the detection of viral RNA via WGS and MinION sequencing in this study was not accompanied by absolute quantification (e.g., genome copies per liter) or threshold cycle (Cq) values derived from calibrated RT-qPCR assays. Without standard curves and quantification controls, the sequencing data reflect presence/absence only, and the number of reads or genome coverage percentage does not necessarily correlate with meaningful viral loads.

Nevertheless, regarding the prevalence of DENV in wastewater samples observed in this study, using genomic sequencing, 23.8% of samples (15 of 63 samples) were similar to those reported in previous studies [18,19]: 21% (24/112 samples) and 25% (68/273), respectively, conducted in Portugal [18] and the United States [19] using RT-PCR. Our future studies will include quantitative RT-qPCR with external RNA standards and primers developed and tested for environmental matrices to determine viral load levels (genome copies/mL) in wastewater samples and assess their public health significance. This is the limitation of the present study; we did not quantify arboviruses’ RNA, because one of the goals was to test and evaluate the feasibility of using the clinical kits commonly applied in public health labs in Brazil for wastewater samples.

From a cost–benefit perspective, our findings underscore that RT-qPCR assays, although inexpensive, rapid, and widely available across public health laboratories, are not suitable for wastewater matrices, resulting in false positives for CHIKV and ZIKV and no detection for DENV. In contrast, sequencing-based approaches (hybrid capture WGS and MinION) proved more reliable for arbovirus detection in environmental samples but required significantly higher costs, advanced infrastructure, and longer turnaround times. Therefore, an integrated approach may offer a practical balance, where RT-qPCR assays optimized for environmental matrices could serve as an initial, low-cost screening tool, followed by sequencing for confirmatory analyses and genetic characterization. Such a combined framework could enhance surveillance while maintaining cost-effectiveness and scalability.

An important limitation of this study is the absence of a systematic evaluation of potential inhibitors commonly present in wastewater, such as humic acids, detergents, and heavy metals. These substances are known to interfere with nucleic acid extraction and enzymatic amplification, leading to false negatives or reduced sensitivity in RT-qPCR assays. Although our sequencing approaches demonstrated higher robustness in detecting arboviruses, the role of inhibitors cannot be excluded and may explain, at least in part, the poor performance of RT-qPCR in this study. Future work should incorporate inhibitor detection assays and spiking experiments with exogenous controls to better quantify the impact of inhibitory compounds in arbovirus wastewater surveillance.

## 4. Conclusions

This study shows that genomic approaches, specifically hybrid-capture whole-genome sequencing (WGS) and MinION nanopore sequencing, can be effective for detecting arboviruses such DENV, CHIKV, and ZIKV in wastewater from both hospital and community settings. However, their apparent superiority over RT-qPCR (using clinical kits) in this context likely reflects methodological differences, including the use of probe-based enrichment and high nucleic acid input volumes, rather than inherent analytical robustness. Furthermore, despite their sensitivity, sequencing-based approaches remain limited by high cost, infrastructure requirements, and turnaround time, which may constrain their routine applicability in environmental surveillance systems.

Our study demonstrated that RT-qPCR assays designed for clinical diagnostics, such as the Trioplex (CDC) and ZDC (IBMP) kits, which are standard across Brazilian public health laboratories during Dengue and Chikungunya outbreaks, are not directly suitable for wastewater samples, likely due to issues with matrix interference, primer specificity, and inadequate validation for environmental conditions. Thus, it is recommended to adapt clinical protocols (kits) for non-conventional matrices to allow WES to be performed by public health laboratories. Rather than discarding RT-qPCR entirely, these findings underscore the need for tailored protocols, including optimized primers and controls, for environmental matrices. Future research should focus on implementing quantitative viral load measurements (e.g., genome copies/mL), expanding longitudinal wastewater monitoring across multiple sites, and assessing the cost-effectiveness and scalability of genomic surveillance approaches for integration into routine public health surveillance programs.

The detection of arboviruses in hospital wastewater underscores its potential as a sentinel surveillance tool, providing an early signal of viral circulation at the community level. Integrating genomic wastewater surveillance into routine monitoring could complement clinical case reporting, enabling earlier public health responses and targeted vector control efforts. Overall, our findings emphasize that a hybrid environmental surveillance strategy using adapted RT-qPCR assays for routine, cost-effective screening and sequencing-based methods as confirmatory tools for pathogen characterization and thus establishing early outbreak signals of arboviruses.

## Figures and Tables

**Table 1:** Detection frequency of DENV, CHIKV, and ZIKV by, WGS, MinION, and RT-PCR.

Sample Type	WGS + MinION			RT-PCR *		
	DENV	CHIKV	ZIKV	DENV	CHIKV	ZIKV
WWTP A	3/18 = 16.7%	4/18 = 22.2%	0/17	ND	1/18 = 5.5%	10/18 = 55.5%
WWTP B	3/15 = 20.0%	2/15 = 13.3%	0/15	ND	ND	9/15 = 60.0%
WWTP C	1/2 = 50.0%	1/2 = 50.0%	0/2	ND	ND	1/2 = 50.0%
Hospital A	5/14 = 35.5%	3/14 = 21.4%	1/14 = 7.1%	ND	1/14 = 7.1%	12/14 = 85.7%
Hospital B	3/14 = 21.4%	2/14 = 14.3%	0/14	ND	1/14 = 7.1%	9/14 = 64.3%
Total WWTP	7/35 = 20%	7/35 = 20%	0/35 = 0%	ND	1/35 = 2.8%	20/35 = 57.1%
Total Hospital	8/28 = 28.6%	5/28 = 17.8%	1/28 = 3.6%	ND	2/28 = 7.1%	21/28 = 75.0%
**Total**	**15/63 = 23.8%**	**12/63 = 19.0%**	**1/63 = 1.6%**	**ND**	3/63 = 4.7% **	41/63= 65.1% ***

* RT-qPCR using the Trioplex (CDC) kit for DENV, CHIKV, and ZIKV, kit ZDC (IBMP) and in-house primers; results are from all 3 assays (kits) combined. ** amplified fragments were sequenced and no CHIKV sequences were retrieved. *** amplified fragments were sequenced and no ZIKV sequences were retrieved. MinION sequencing was not performed for ZIKV. ND not detected.

**Table 2:** Detection frequency of DENV1 by WGS, MinION, RT-qPCR vs. Weekly Reported Cases.

Sample ID/Date	WGS (N. Reads/% Coverage)	MinION	RT-qPCR *	Dengue Cases **
47-HA (07/23/22)	42/9.0%	ND	-	31
49-HA (08/12/22)	ND	ND	-	23
50-HB (08/12/22)	30/4.3%	ND	-	23
51-HA (09/01/22)	14/3.2%	NT	-	19
52-HB (09/01/22)	ND	ND	-	19
54-WWA (02/08/23)	ND	ND	-	161
55-HA (02/08/23)	8/3.9%	NT	-	161
56-WWB (02/08/23)	50/9.5%	NT	-	161
59-WWA (02/23/23)	ND	NT	-	267
61-HA (02/23/22)	10/3.3%	NT	-	267
65-WWA (03/01/23)	68/9.4%	5863/80.1%	-	430
66-WWA (03/01/23)	10/3.3%	ND	-	430
67-HA (03/01/23)	12/4.5%	ND	-	430
68-WWB-I (03/01/23)	82/7.2%	ND	-	430
69-WWB-E (03/01/23)	61/5.5%	ND	-	430
70-HB (03/01/23)	97/13.1%	ND	-	430
71-WWA (03/15/23)	12/5.0%	NT	-	604
72-WWA (03/15/23)	28/1.3%	NT	-	604
74/75-WWB (03/15/23)	ND	ND	-	604
77-WWC-I (03/15/23)	34/10.9%	NT	-	604
78-WWC-E (03/15/23)	ND	ND	-	604
79-WWA (02/23/23) ^1^	2/1.36%	3/79.3%	-	267
81-HA (02/23/22) ^2^	8/1.22%	NT	-	267
82-WWA (02/23/23) ^1^	12/4.6%	NT	-	267
62/83-WWB (02/23/23)	70/3.99%	ND	-	267
84-HA (02/24/23) ^2^	ND	NT	-	267
87/34-HB (12/01/22)	2/1.33%	NT	-	22
320-HB (03/14/23)	ND	NT	-	604
323-HA (04/11/23)	ND	NT	-	1415
329-WWA (05/2023)	ND	NT	-	883
**Total Detection**	**20/30 = 66.6%**	**2/15 = 13.3%**	**0%**	

ND: not detected (genome not present). NT: sample was not tested; ^1^ 59/79/82: same sample. ^2^ 84/81/61: same sample; * RT-qPCR was performed using the Trioplex CDC kit (positive amplification defined as Cq < 38), ZDC IBMP kit (positive defined as Cq < 36), and with in-house primers for subtypes 1, 2, 3, and 4. ** Number of Dengue cases in Belo Horizonte according to [31,32]. WW-I: influent sample from WWTP A, B, and C, WW-E: effluent sample.

**Table 3:** Detection of CHIKV by WGS, MinION, RT-qPCR vs. Weekly Reported Cases.

Sample ID (Month/Day/Year)	WGS	MinION (N. Reads/Coverage)	RT-qPCR * (Cq Number)	Chikungunya Cases **
47-HA (07/23/22)	ND	32,123/7.2%	-	3
49-HA (08/12/22)	ND	477/25.5%	-	6
50- HB (08/12/22)	ND	ND	-	6
51- HA (09/01/22)	ND	NT	-	5
52- HB (09/01/22)	ND	2853/17.7%	-	5
54-WWA (02/08/23)	ND	84/61.8%	-	117
55- HA (02/08/23)	ND	NT	-	117
56-WWB (02/08/23)	ND	NT	-	117
59- WWA (02/23/23) ^1^	ND	NT	-	148
61-HA (02/23/22) ^2^	ND	NT	-	148
65- WWA (03/01/23)	ND	1781/12.8%	-	253
66- WWA (03/01/23)	ND	22,640/33.4%	-	253
67- HA (03/01/23)	ND	19,788/6.5%	-	253
68-WWB (03/01/23)	ND	NT	-	253
69-WWB (03/01/23)	61/2.8%	84/40.4%	-	253
70- HB (03/01/23)	ND	5423/26.4%	-	253
71-WWA (03/15/23)	ND	NT	-	414
72-WWA (03/15/23)	ND	NT	-	414
74/75-WWB (03/15/23)	ND	ND	-	414
77-WWC-I(03/15/23)	ND	NT	-	414
78-WWC-E(03/15/23)	ND	63/19.6%	-	414
79-WWA (02/23/23) ^1^	76/6.8%	70/7.4%	-	148
81-HA (02/23/23) ^2^	ND	NT	-	148
82-WWA (02/23/23) ^1^	ND	NT	-	148
62/83-WWB (02/23/23)	ND	26,495/15.4%	-	148
84-HA (02/24/23) ^2^	ND	NT	-	148
87/34-HB (12/01/22)	ND	NT	-	1
320-HB (03/14/23)	ND	NT	+(27.0) ***	414
323-HA (04/11/23)	ND	NT	+(29.0) ***	719
329-WWA (05/10/23)	ND	NT	+(28.0) ***	479
**Total detection**	**2/30 = 6.7%**	**12/14 = 85.7%**	**3/30 = 10%**	

ND: not detected (genome not present); NT: sample was not tested; ^1^ 59/79/82: same sample; ^2^ 61/81/84: same sample; * RT-qPCR was performed using the Trioplex CDC kit (positive defined as Cq < 38) and in-house primers. ** Number of cases in Belo Horizonte according to [32,33]. *** RT-qPCR amplified fragments (Trioplex CDC kit) were sequenced and no CHIKV sequences were retrieved.

**Table 4:** Detection frequency of ZIKV in Wastewater by WGS and RT-qPCR.

Sample ID/Date	WGS	RT-qPCR (Cq Number)
47-HA (07/23/22)	ND	+(29.0)
49-HA (08/12/22)	ND	+(26.6)
50-HB (08/12/22)	ND	−
51-HA (09/01/22)	ND	+(34.0) *
52-HB (09/01/22)	ND	+(26.9)
54-WWA (02/08/23)	ND	+(26.8)
55-HA (02/08/23)	215 reads/1.40% coverage	+(34.7) *
56-WWB (02/08/23)	ND	+(35.8) *
59-WWA (02/23/23) ^1^	ND	+(37.6)
61-HA (02/24/23) ^2^	ND	− *
65-WWA (03/01/23)	ND	+(24.6)
66- WWA (03/01/23)	ND	+(25.9) **
67- HA (03/01/23)	ND	+(27.4)
68- WWB (03/01/23)	ND	+(21.3)
69- WWB (03/01/23)	ND	+(25.4)
70- HB (03/01/23)	ND	+(26.8)
71-WWA (03/15/23)	ND	NT
72-WWA (03/15/23)	ND	− *
74/75-WWB (03/15/23)	ND	+(28.0)
77- WWC-I (03/15/23)	ND	− *
78-WWC-E (03/15/23)	ND	+(21.0)
79-WWA (02/23/23) ^1^	ND	+(19.8)
81-HA (02/24/23) ^2^	ND	−
82-WWA (02/23/23) ^1^	ND	NT
62/83-WWB (02/23/23)	ND	+(20.7)
84-HA (02/24/23) ^2^	ND	+(31.3) *
87/34-HB (12/01/22)	ND	− *
320-HB (03/14/23)	ND	+(23.0) ***
323-HA (04/11/23)	ND	+(26.0) ***
329-WWA (05/2023)	ND	+(23.0) ***
**Detection**	**1/30 = 3.3%**	**22/28 = 78.6% (false positive)**

ND: not detected (genome not present). NT: sample was not tested. ^1^ 59/79/82 same sample; ^2^ 61/81/84, same sample; * RT-qPCR was performed using the ZDC (IBMP) kit, positive amplification defined as Cq < 36. For other samples RT-qPCR was performed using the Trioplex CDC kit (positive defined as Cq < 38) and with in-house primers; ** fragment was sequenced and retrieved sequences of *Sulfurospirillum* bacteria. *** fragments were sequenced and no ZIKV sequences were retrieved.

## Data Availability

The sequences generated in this study were deposited in GenBank (https://www.ncbi.nlm.nih.gov/bioproject/PRJNA1328205, accessed on 13 August 2025) under the sample codes SAMN42174356 to SAMN42174437. Other results are original contributions and are included in the article. Further inquiries can be directed to the corresponding author.

## Abbreviations

The following abbreviations are used in this manuscript:

RT-qPCR	reverse transcription quantitative polymerase chain reaction
WGS	Whole Genome Sequencing
WWTP	Waste Water Treatment Plant
